# Figainin 1, a Novel Amphibian Skin Peptide with Antimicrobial and Antiproliferative Properties

**DOI:** 10.3390/antibiotics9090625

**Published:** 2020-09-21

**Authors:** Carlos José Correia Santana, Ana Carolina Martins Magalhães, Agenor C. M. dos Santos Júnior, Carlos André Ornelas Ricart, Beatriz D. Lima, Alice da Cunha Morales Álvares, Sonia Maria de Freitas, Osmindo Rodrigues Pires, Wagner Fontes, Mariana S. Castro

**Affiliations:** 1 Laboratory of Toxinology, Department of Physiological Sciences, Institute of Biology, University of Brasília, Brasília 70.910-900, DF, Brazil; carlosjcsantana@gmail.com (C.J.C.S.); bioana.11@gmail.com (A.C.M.M.); osmindo@unb.br (O.R.P.J.); 2 Laboratory of Protein Chemistry and Biochemistry, Department of Cell Biology, Institute of Biology, University of Brasília, Brasília 70.910-900, DF, Brazil; agenor.unb@gmail.com (A.C.M.d.S.J.); ricart@unb.br (C.A.O.R.); wagnerf@unb.br (W.F.); 3 Laboratory of Gene Biology, Department of Cell Biology, Institute of Biology, University of Brasília, Brasília 70.910-900, DF, Brazil; beatrizdolabela@unb.br; 4 Laboratory of Biophysics, Department of Cell Biology, Institute of Biology, University of Brasília, Brasília 70.910-900, DF, Brazil; pharmalice@gmail.com (A.d.C.M.Á.); nina@unb.br (S.M.d.F.)

**Keywords:** amphibian, *Boana raniceps*, skin secretion, structural analysis, antimicrobial peptide, cytolytic peptide, hemolysis

## Abstract

Amphibian skin secretions are abundant in bioactive compounds, especially antimicrobial peptides. These molecules are generally cationic and rich in hydrophobic amino acids, have an amphipathic structure and adopt an α-helical conformation when in contact with microorganisms membranes. In this work, we purified and characterized Figainin 1, a novel antimicrobial and antiproliferative peptide from the cutaneous secretion of the frog *Boana raniceps*. Figainin 1 is a cationic peptide with eighteen amino acid residues—rich in leucine and isoleucine, with an amidated C-terminus—and adopts an α-helical conformation in the presence of trifluoroethanol (TFE). It displayed activity against Gram-negative and especially Gram-positive bacteria, with MIC values ranging from 2 to 16 µM, and showed an IC_50_ value of 15.9 µM against epimastigote forms of *T. cruzi*; however, Figanin 1 did not show activity against *Candida* species. This peptide also showed cytolytic effects against human erythrocytes with an HC_50_ of 10 µM, in addition to antiproliferative activity against cancer cells and murine fibroblasts, with IC_50_ values ranging from 10.5 to 13.7 µM. Despite its adverse effects on noncancerous cells, Figainin 1 exhibits interesting properties for the development of new anticancer agents and anti-infective drugs against pathogenic microorganisms.

## 1. Introduction

Amphibian skin is a highly specialized structure that is exposed to the environment and consequently to microorganisms and parasites [1]. To cope with these adverse conditions, amphibian skin is rich in glands that secrete a wide variety of biologically active compounds such as biogenic amines, steroids, alkaloids, bufodienolides, peptides and proteins. These molecules exhibit various biological activities including neurotoxic, vasoconstrictive, hallucinogenic, hypotensive, antimicrobial and cytotoxic properties [2,3]. Among these molecules, antimicrobial peptides (AMPs) have great therapeutic potential due to their cytotoxic capacity. AMPs are characterized by having a relatively short chain (10–50 amino acid residues), are generally positively charged (+2 to +9) and show a high percentage of hydrophobic amino acids (≥30%) [4]. These properties allow these peptides to adopt an amphipathic conformation when in contact with the microorganisms’ plasma membrane [4,5]. Due to the growing number of microorganisms resistant to conventional antibiotics and the need for new anti-infective drugs for human therapy, AMPs stand out for presenting a broad spectrum in addition to a low propensity for resistance development [6,7].

From the skin secretions of different species of the genus *Hypsiboas*, currently reclassified as *Boana* [8], several broad-spectrum antimicrobial peptides have been isolated [9,10,11,12]. Our research group originally found a new class of peptides named Hylins from the skin secretion of *Hyla biobeba* (now *Boana lundii*) with hemolytic activity [13] and, later, Hylin a1 from *Hypsiboas albopunctatus* (now *Boana albopunctata*) with a broad spectrum of activity against Gram-positive and Gram-negative bacteria and fungi, in addition to strong hemolytic activity [14].

In this work, we report the isolation and chemical and biological characterization of a new antimicrobial and anticancer peptide from the skin secretion of the anuran *Boana raniceps* (Cope, 1862).

## 2. Material and Methods

### 2.1. Obtention of Skin Secretion

Adult individuals of *B. raniceps* were collected in Monte Alegre de Goiás (Goiás, Brazil) during the night, and the skin secretion extraction was performed by mild electrical stimulation for 10–20 s, with a pulse duration of 3 ms, at 0 to 50 V (with the voltage adjusted by a potentiometer), at 60 Hz. The skin secretion was dissolved in Milli-Q water, frozen, lyophilized and stored at −20 °C until use. The specimens were returned to their original environment after extraction. Collection was carried out under the license number 51541-1 from the Brazilian Environmental Agency (IBAMA-SISBIO), and the studies were performed under the protocol number AA272A7 from SisGen (National System for the Management of Genetic Heritage and Associated Traditional Knowledge). The skin-secretion-harvesting procedure was approved by the Animal Ethics Committee of the University of Brasília.

### 2.2. Peptide Purification

Aliquots containing 8.0 mg of *B. raniceps* skin secretion were dissolved in 1 mL of trifluoroacetic acid (TFA) 0.1% (*v*/*v*) in Milli-Q water (solution A) and centrifuged for 10 min at 13,800× *g*, and then, 200 µL of the supernatant was injected into a C_8_ reversed-phase HPLC analytical column (Vydac 208TP54, 4.6 × 250 mm, Grace, CA, USA) coupled to a LC-20AT Prominence Liquid Chromatograph (Shimadzu, Kyoto, Japan). Chromatographic runs were executed using a column previously equilibrated with solution A, and separation was performed by applying an optimized gradient of solution B (TFA 0.1% (*v*/*v*) in acetonitrile).

The rechromatography step applied for the purification of the antimicrobial fraction was performed using the same chromatographic system and the same mobile phases on a C_18_ column (Shim-pack VP-ODS, 4.6 × 150 mm, Shimadzu, Kyoto, Japan).

All runs were performed at a flow rate of 1 mL/min, with UV detection at 216 nm and at room temperature (22 ± 2 °C). The eluted fractions were manually collected, lyophilized and kept at −20 °C until use.

### 2.3. Peptide Quantification

The peptide stock solution was quantified according to the methodology described by Aitken and Learmonth [15]. Aliquots of Figainin 1 at 128 µM were prepared, lyophilized and kept at −20 °C until the moment of use. Each aliquot was resuspended in medium or buffer according to the protocol of each experiment, maintaining the desired concentration as determined by peptide quantification.

### 2.4. Structural Characterization

#### 2.4.1. MALDI-TOF MS Analysis and N-Terminal Chemical Sequencing

The molecular mass of the purified peptide was determined by matrix-assisted laser desorption/ionization–time of flight mass spectrometry (MALDI-TOF MS) using a Bruker Autoflex II TOF/TOF instrument (Bruker Daltonics, Bremen, Germany). One microliter of peptide solubilized in acetonitrile/water (1:1, *v*/*v*) containing TFA 0.1% (*v*/*v*) was added to a stainless-steel plate and mixed with 1 µL of α-cyano-4-hydroxycinnamic acid (HCCA, 10 mg/mL). The analysis was performed using reflected positive mode in the *m*/*z* range of 550–4000 in a previously calibrated spectrometer (using Peptide Calibration Standard II, Bruker Daltonics, Bremen, Germany).

The presence of C-terminal amidation in the purified peptide was evaluated by mass spectrometry analysis of the methylated peptide according to the methodology described by Hunt et al. [16]. Ten micrograms (10 µg) of the peptide was solubilized in methanolic HCl reagent (10 µL of acetyl chloride in 250 µL of distilled methanol); after 1 h at room temperature, the solvent was removed in a vacuum concentrator and the sample was analyzed by MALDI-TOF MS as described above.

The primary structure of the peptide was obtained by automatic Edman degradation using a Shimadzu PPSQ-33A protein sequencer (Shimadzu, Kyoto, Japan).

#### 2.4.2. Secondary Structure Analysis by Circular Dichroism

Figainin 1’s secondary structure components were assessed by circular dichroism (CD) analysis in a Jasco J-815 spectropolarimeter 113 (Jasco, Tokyo, Japan). The measurements were performed at 25 °C, with the temperature controlled by a Peltier system. CD spectra were obtained using a 0.1 cm pathlength quartz cuvette in the wavelength range 190–260 nm and data acquisition at 0.2 nm. The peptide was dissolved in water and 10%, 30% and 50% (*v*/*v*) trifluoroethanol (TFE). The final spectra were obtained by the accumulation of four consecutive measurements, and the mean spectra were corrected for the baseline contribution of water and TFE solutions. The observed ellipticities were converted to molar ellipticity ([θ]) (deg·cm^2^·dmol^−1^) based on a mean molecular mass per residue of 115 Da, and the α-helix secondary structure content was estimated considering the values of [θ]_208 nm_ [17].

#### 2.4.3. Bioinformatics Analysis

The amino acid sequence of the peptide was queried against the nr NCBI database using BLAST (http://blast.ncbi.nlm.nih.gov/Blast.cgi) [18] and against the Antimicrobial Peptide Database (APD, http://aps.unmc.edu/AP/database/query_input.php) [19] for similarity searches. Clustal Omega [20] was used to perform multiple sequence alignments; EMBOSS Needle—Pairwise Sequence Alignment (https://www.ebi.ac.uk/Tools/psa/emboss_needle/) was used for global alignment of two sequences; the Expasy Compute pI/Mw tool (http://web.expasy.org/compute_pi) [21] was used for theoretical molecular mass calculation. The calculation of the GRAVY (Grand Average of Hydropathicity) value was performed using the ProtParam tool (https://web.expasy.org/protparam/) [22], and the NetWheels software (https://github.com/molx/NetWheels) [23] was used to plot a helical wheel representation of Figainin 1.

### 2.5. Antibacterial and Antifungal Assays

After the chromatographic fractionation of the *B. raniceps* skin secretion, the fractions were subjected to preliminary assays against *Staphylococcus aureus* (ATCC 25923) and *Escherichia coli* (ATCC 25922) in order to identify the antimicrobial fractions. Each fraction was incubated with the bacterial suspensions prepared as described below, incubated for 22 h at 37 °C, and bacterial growth was determined by optical density (OD) measurement at 595 nm using a microplate reader (Multiskan FC, Thermo Scientific, San Jose, CA, USA).

The minimal inhibitory concentration (MIC) for pathogenic bacteria and fungi were determined as described in [14]. The following pathogenic microorganisms were used: the Gram-positive bacteria *Enterococcus faecalis* (ATCC 29212), *Staphylococcus aureus* (ATCC 25923), *Staphylococcus epidermidis* (ATCC 12228) and *Enterobacter casseliflavus* (ATCC 700327); the Gram-negative bacteria *Escherichia coli* (ATCC 25922), *Klebsiella pneumoniae* (ATCC 13883) and *Pseudomonas aeruginosa* (ATCC 27853); and the yeasts *Candida albicans* (ATCC 90028) and *Candida parapsilosis* (ATCC 22019). The bacteria were grown overnight in Mueller–Hinton (MH) broth, and the yeasts were grown overnight in Brain Heart Infusion (BHI) broth at 37 °C under agitation. Then, the OD was adjusted to 1.0 at 590 nm, and the suspensions were diluted in the respective medium (1:100 for Gram-positive bacteria and fungi and 1:50 for Gram-negative bacteria) and incubated in the presence of Figainin 1 at different concentrations for 22 h at 37 °C. For MIC assessment, dilutions of the peptide were made to obtain concentrations of 64, 32, 16, 8, 4, 2, 1 and 0.5 μM. After the incubation time, the OD values of the wells were determined at 595 nm in a Multiskan FC microplate reader (Thermo Scientific, San Jose, CA, USA). Milli-Q water, formaldehyde 0.8% (*v*/*v*) and the antibiotics ampicillin (Sigma-Aldrich, St. Louis, MO, USA) and vancomycin (Sigma-Aldrich, St. Louis, MO, USA) were used as controls. Each assay was performed in triplicate. The minimal inhibitory concentration (MIC) was defined as the lowest peptide concentration at which the visible growth of the microorganism tested was completely inhibited.

### 2.6. Anti-Epimastigote Activity against Trypanosoma cruzi

Epimastigote forms of *T. cruzi* (CL-Brener strain) were grown in Liver Infusion Tryptose (LIT) medium supplemented with 10% fetal bovine serum (FBS) at 28 °C. Then, parasite suspension containing 5 × 10^6^ parasites/mL was incubated in a 96-well microplate for 48 h in the absence or presence of Figainin 1 at different concentrations dissolved in LIT medium. For anti-*T. cruzi* activity assessment, dilutions of the peptide were made to obtain concentrations of 64, 32, 16, 8, 4, 2, 1, 0.5 and 0.25 µM. Parasite viability was determined using the CellTiter-Blue^®^ Cell Viability Assay (Promega, Madison, WI, USA) protocol. The cells were incubated for 4 h at 37 °C prior to recording fluorescence (560_Ex_/590_Em_) in a SpectraMax microplate reader (Molecular Devices, San Jose, CA, USA). The IC_50_ value was determined using the GraphPad Prism (version 5.04) program.

### 2.7. Hemolytic Assay

The hemolytic activity of Figainin 1 was evaluated by determining hemoglobin release from human red blood cells (hRBCs) suspensions. Fresh hRBCs obtained from a healthy donor were washed three times using a Tris-saline buffer (0.01 M Tris–HCl, pH 7.4, containing 0.15 M NaCl). Then, a 1% (*v*/*v*) suspension of hRBCs was prepared in Tris-saline. Aliquots of 100 µL of a 1% (*v*/*v*) suspension of hRBCs were directly added to 100 µL of Figainin 1 at different concentrations. For hemolytic activity assessment, dilutions of the peptide were made to obtain concentrations of 64, 32, 16, 8, 4, 2, 1, 0.5 and 0.25 µM. All samples were incubated for 1 h at room temperature. After centrifugation at 400× *g* for 5 min, the absorbance of the supernatants was measured at 405 nm using a Multiskan FC microplate reader (Thermo Scientific, San Jose, CA, USA).

Tris-saline buffer and 1% (*v*/*v*) Triton X-100 mixed with a 1% (*v*/*v*) suspension of hRBCs were used as negative and positive controls. The assays were performed in triplicate, and the data were expressed as mean ± SD. Hemolytic activity was calculated using the following formula: % hemolysis = 100 × (A_peptide_−A_Tris-saline_)/(A_triton_−A_Tris-saline_), where A_peptide_ was the absorbance of the release of hemoglobin in the presence of Figainin 1; A_Tris-saline_, that for the buffer; and A_triton_, that for the Triton X-100 positive control. The peptide concentration that caused 50% lysis of hRBCs (HC_50_) was calculated by nonlinear regression using the GraphPad Prism (version 5.04) software. The hRBC harvesting procedure was approved by the Human Ethics Committee of the University of Brasília.

### 2.8. Antiproliferative Assay

The antiproliferative activity of Figainin 1 was evaluated in mouse embryonic fibroblasts NIH/3T3 (ATCC CRL-1658) and three different cancer cell lines: murine skin melanoma B16F10 (ATCC CRL-6475) cells, human mammary adenocarcinoma MCF-7 (ATCC HTB-22) cells and human cervical adenocarcinoma HeLa (ATCC CCL-2) cells.

The cells were maintained as described by [24]. Briefly, all the cell lines were seeded in 75 cm^2^ culture flasks containing complete medium (Dulbecco’s modified Eagle’s medium supplemented with 10% fetal bovine serum, 100 IU/mL penicillin and 100 µg/mL streptomycin) and maintained in an incubator at 37 °C in 5% CO_2_. Cells were seeded at a density of 8 × 10^3^/well for MCF-7 and HeLa and 5 × 10^3^/well for B16F10 and mouse fibroblasts NIH/3T3 in 96-well microplates with complete medium overnight. Then, the cells were treated with Figainin 1 at different concentrations and incubated for 24 h at 37 °C in 5% CO_2_. For antiproliferative activity assessment, dilutions of the peptide were made to obtain concentrations of 64, 32, 16, 8, 4, 2, 1 and 0.5 µM. After the incubation period, aliquots of 15 µL of 3-(4,5-dimethylthiazol-2-yl)-2,5-diphenyltetrazolium bromide (MTT, Molecular Probes, Thermo Fisher Scientific, Oregon, USA) at 5 mg/mL in PBS, pH 7.4, and 135 µL of complete medium were added to the wells and incubated for 3 h. The formazan crystals were solubilized by the addition of 100 µL of dimethyl sulfoxide (DMSO), and the absorbance signal at 595 nm was measured using a Multiskan FC microplate reader (Thermo Scientific, San Jose, CA, USA). The half maximal inhibitory concentration (IC_50_) was calculated using the GraphPad Prism (version 5.04) software.

## 3. Results

### 3.1. *Isolation, Identification and Structural Characterization of Figainin 1*

**Figure 1:**
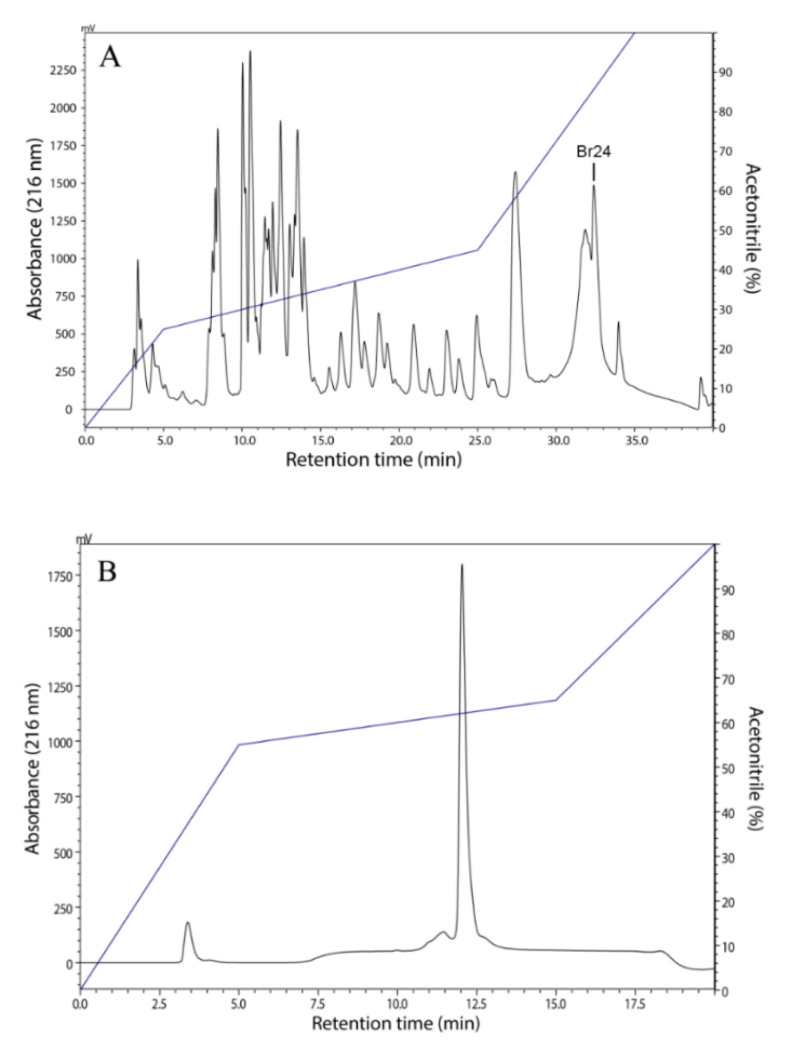
(**A**) Reversed-phase HPLC profile of *B. raniceps* skin secretion fractionated on a Vydac C_8_ column. One antibacterial fraction is indicated as Br24. The blue line shows the concentration of solution B (acetonitrile + TFA 0.1%, *v/v*). (**B**) Purification of the antimicrobial peptide (present in Br24 fraction) on a Shim-pack VP-ODS C_18_ column.

The chromatographic fractionation of the *B. raniceps* skin secretion was performed using an analytical C_8_ column (Figure 1A). Aliquots of the eluted factions were tested for their ability to inhibit the growth of the pathogenic bacterial *E. coli* (ATCC 25922) and *S. aureus* (ATCC 25923). Among the active fractions; one (named Br24) was selected, accumulated and purified to homogeneity by RP-HPLC using a C_18_ column as shown in Figure 1B.

**Figure 2:**
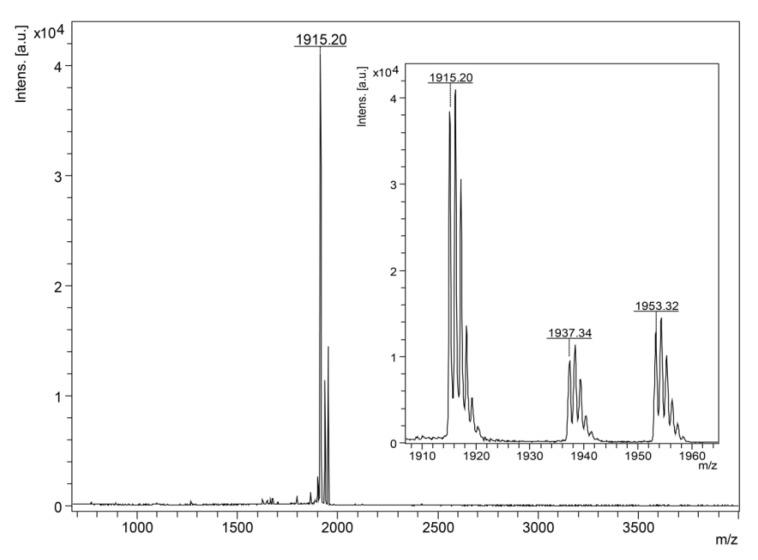
MALDI-TOF mass spectrum of the antibacterial peptide (isolated from Br24 fraction). In the insert is shown the presence of sodium (+22 Da) and potassium (+38 Da) adducts.

The antibacterial peptide, after purification, was analyzed by MALDI-TOF MS and showed one component with a monoisotopic molecular mass [M + H]^+^ of 1915.20 Da (Figure 2). Its primary structure was determined by Edman degradation and resulted in an unambiguous sequence: ^1^FIGTLIPLALGALTKLFK^18^. Since the difference of 1 Da between the theoretical and the observed molecular mass indicates the possible presence of a C-terminal amidation, Fischer esterification of the peptide was performed, confirming the presence of this type of post-translational modification (data not shown).

**Figure 3:**
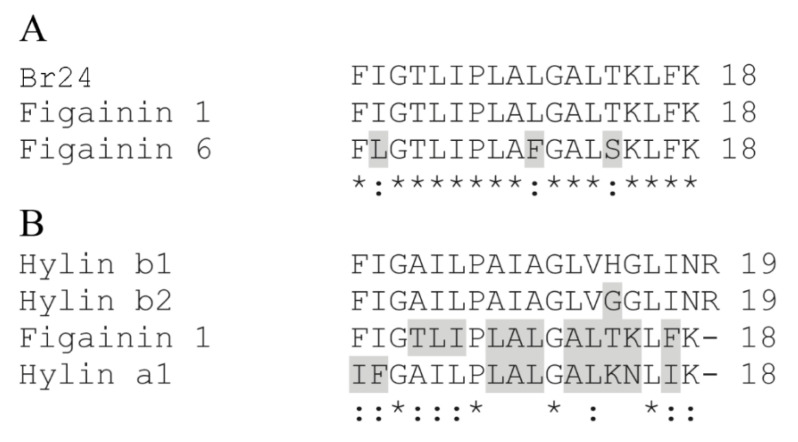
(**A**) Sequence alignment of the antibacterial peptide (isolated from Br24 fraction) with other putative antimicrobial peptides from *B. raniceps* [25] leading to the identification of Figainin 1, and (**B**) sequence alignment of Figainin 1 with Hylins (antimicrobial peptides isolated from other species in *Boana* genus). Differences of amino acids are highlighted in gray, ‘*’ indicates identical amino acids and ‘:’ indicates conservative substitutions.

Similarity searches using nr BLAST and the APD were carried out based on the amino acid sequence of the purified peptide and revealed that this peptide corresponds to Figainin 1 and exhibits high similarity (94.4%) to Figainin 6, both putative peptides derived from a cDNA library constructed from *B. raniceps* skin [25] (Figure 3A). When compared to the cytolytic peptides Hylins isolated from other species of the *Boana* genus, Figainin 1 is more similar to Hylin a1 with 66.7% of similarity and 55.6% of identity (Figure 3B).

Figainin 1 is cationic with a net charge of +3 and is rich in hydrophobic amino acids (61%) with a GRAVY value of 1.46 (Table 1), indicating a hydrophobic character for this peptide.

**Figure 4:**
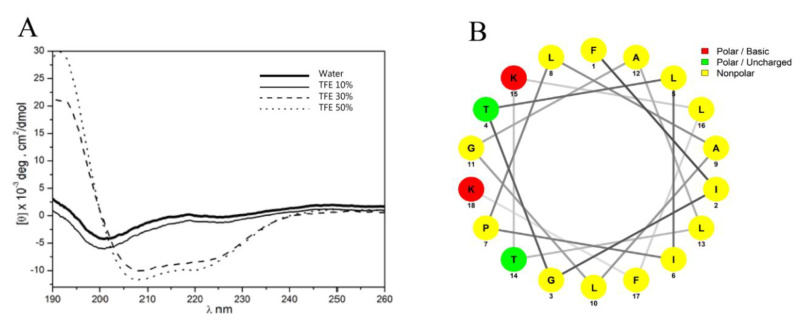
(**A**) Circular dichroism spectra of Figainin 1 dissolved in water and in crescent concentrations (10, 30 and 50%, *v/v*) of trifluoroethanol (TFE). (**B**) Helical wheel representation of Figainin 1 illustrating the amphipathic character of the putative α-helix.

Figainin 1’s secondary structure analysis was performed by circular dichroism (CD) in water and in crescent concentrations (10, 30 and 50%, *v/v*) of trifluoroethanol (TFE) (Figure 4A). In water and 10% (*v/v*) TFE, Figainin 1 showed a mostly disordered structure, whereas in the presence of 30% (*v/v*) and 50% (*v/v*) TFE, this peptide showed α-helical content of 62 and 69%, respectively, characterized by the presence of negative dichroic bands centered at 208 and 222 nm. The helical wheel representation indicates that Figainin 1 adopts an amphipathic α-helix conformation with a wide hydrophobic face rich in leucine and isoleucine amino acids (Figure 4B).

### 3.2. Antimicrobial and Antitrypanosomal Activity

The inhibitory effect of Figainin 1 against pathogenic microorganisms was evaluated (Table 2), and it exhibited activity against the Gram-negative bacteria *E. coli* (MIC = 16 µM) and *K. pneumoniae* (MIC = 4 µM) but did not show activity against *P. aeruginosa*. Considering Gram-positive microorganisms, Figainin 1 was active against all tested bacteria with MIC values ranging from 2 µM against *S. epidermidis* to 16 µM against *E. casseliflavus*. On the other hand, it showed no activity against the yeasts *C. albicans* and *C. parapsilosis* at a maximum concentration of 64 µM. Figainin 1 also showed activity against epimastigote forms of *T. cruzi*, with IC_50_ = 15.9 µM.

### 3.3. Hemolytic Activity

**Figure 5:**
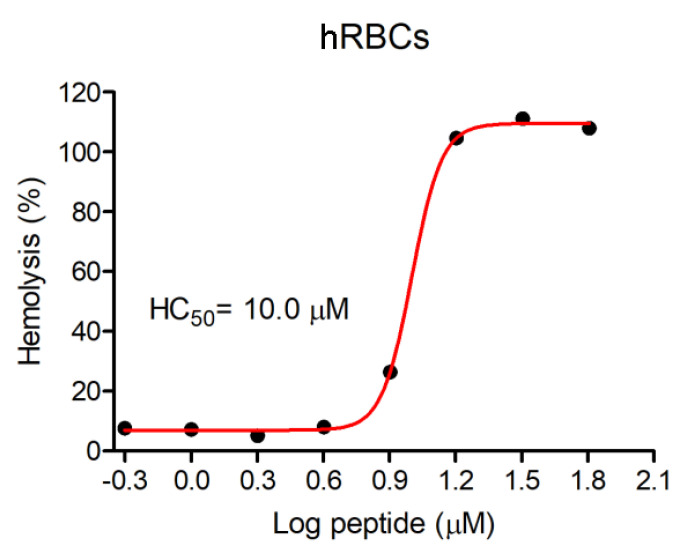
The hemolytic effect of Figainin 1 on human erythrocytes (hRBCs).

Hemolytic activity against human red blood cells (hRBCs) was evaluated, and this assay demonstrated that Figainin 1 exhibited high hemolytic activity, with HC_50_ = 10 µM (0.019 g/L) (Figure 5).

### 3.4. Antiproliferative Activity of Figainin 1

**Figure 6:**
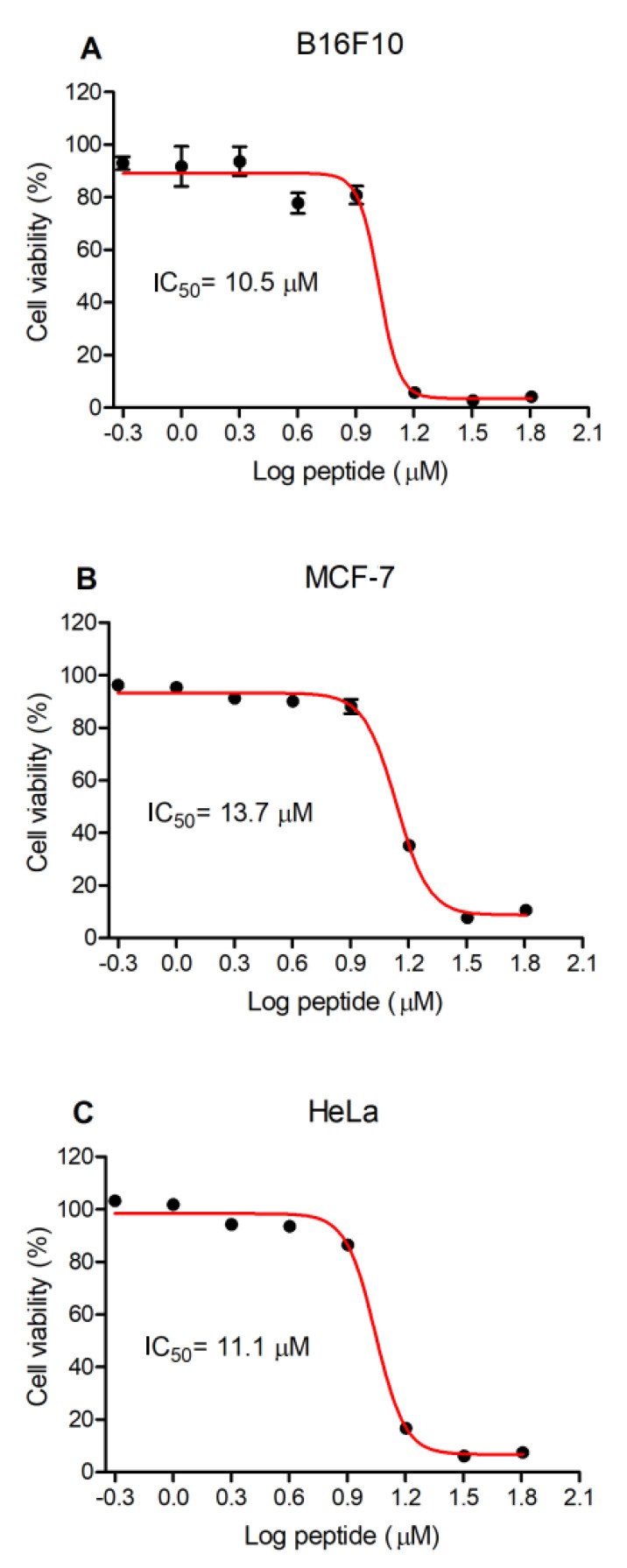
The effect of Figainin 1 on the proliferation of (**A**) murine skin melanoma B16F10 cells, (**B**) human mammary adenocarcinoma MCF-7 cells and (**C**) human cervical adenocarcinoma HeLa cells.

The antiproliferative activity of Figainin 1 against mouse embryonic fibroblasts NIH/3T3 and three different cancer cell lines (the murine skin melanoma cell line B16F10, human mammary adenocarcinoma cell line MCF-7 and human cervical adenocarcinoma HeLa cells) was evaluated using the MTT assay (Figure 6). Similar results were observed among all the tested cell lines: NIH/3T3, IC_50_ = 13 µM (0.025 g/L); B16F10 cells, IC_50_ = 10.5 µM (0.020 g/L) (Figure 6A); MCF-7 cells, IC_50_ = 13.7 µM (0.026 g/L) (Figure 6B); and HeLa cells, IC_50_ = 11.1 µM (0.021 g/L) (Figure 6C).

## 4. Discussion

The amphibian skin secretions are an important source of biologically active molecules that have been extensively studied as therapeutic alternatives for the treatment of several diseases, especially those caused by multi-drug-resistant microorganisms [26,27,28]. Since the isolation of Magainins, a class of antimicrobial peptides isolated from the African clawed frog *Xenopus laevis* by Michael Zasloff [29], more than 1000 antimicrobial peptides or host defense peptides (HDPs) have been isolated from anurans according to the antimicrobial peptide database APD [19].

In contrast to the action of traditional antimicrobial agents that act primarily on bacterial physiological processes such as DNA replication and cell wall synthesis, the action of antimicrobial peptides occurs mainly by direct effects on the bacterial plasmatic membrane without the mediation of specific receptors and can also act on intracellular targets, resulting in protein inhibition or the inhibition of DNA and RNA synthesis or act as an immunomodulator [5,7,30,31].

AMPs are generally amphipathic and positively charged, favoring greater interaction with negatively charged phospholipids such as phosphatidylglycerol, phosphatidylethanolamine and cardiolipin from bacterial membranes than with the “zwiterionic” (neutral) phospholipids present on the extracellular surface of mammalian cell membranes [32]. Differences are observed in the membranes of cancer cells, as a greater amount of negatively charged components such as phosphatidylserine, glycoproteins and glycolipids in addition to differences in membrane fluidity can increase the activity of cytolytic peptides when compared to that for normal cells [33].

In this study, we purified the AMP Figainin 1 from *B. raniceps* skin secretion. The sequence analysis of Figainin 1 showed high similarity to the cytolytic peptide Hylin a1 isolated from *B. albopunctata* and lower similarity to Hylins b1 and b2 from *B. lundii* (*Hyla biobeba*), all C-terminally amidated [13,14]. Hylin a1 is characterized by a wide nonpolar face rich in the hydrophobic amino acids leucine and isoleucine. Its antimicrobial activity is more potent against Gram-positive microorganisms, and it exhibits high hemolytic activity [14]. Similar properties were observed for Figainin 1 with the exception of the antifungal activity, whereas Hylin a1 is active against *Candida* species and *Cryptococcus neoformans*; Figainin 1 did not show activity against *Candida* species.

There is a consensus that the biological activity of peptides as well as their selectivity is due to their physicochemical parameters such as charge, helicity and hydrophobicity, in addition to the sizes of their hydrophilic and hydrophobic domains [34,35]. Figainin 1 exhibited the highest retention time (Figure 1A), and also high hydrophobic ratio (61%) and GRAVY value, as shown in Table 1. The helical wheel representation indicated that Figainin 1 presents a large nonpolar face rich in leucine and isoleucine amino acids (Figure 4B). Chen et al. [36] showed that a high hydrophobicity is correlated with a greater hemolytic activity and Figainin 1 is strongly hemolytic.

C-terminal amidation is one of the most commonly found post-translational modifications in bioactive peptides from different origins [1,37]. It has been reported that amidation results in greater biological activity of peptides, including inhibitory effects against pathogenic bacteria, but also increases deleterious effects such as hemolysis [38]. Using circular dichroism and molecular dynamics simulations, Mura et al. showed that Aurein analogs with C-terminal amidation are more likely to form α-helices than the same peptides in the acidic form [39]. In another study, using the anionic peptide Maximin-H5 as a template, it was reported that the C-terminally amidated peptide was more cytotoxic than the deamidated form, indicating that this modification provides greater stability to the peptide when interacting with membranes [40]. A similar result was observed using the acidic and amidated forms of Protonectarin-MP mastoparan, where the C-terminally amidated peptide had its biological activity potentiated, including hemolytic activity, due to greater stabilization of the secondary structure of the peptide, favoring the α-helix [41].

Figainin 1 showed potent hemolytic activity (Figure 5), in addition to antiproliferative activity, against cancer cells and murine fibroblasts, with IC_50_ values ranging from 10.5 to 13.7 µM (Figure 6). In fact, hemolytic activity and low selectivity are important limiting factors in the therapeutic application of AMPs. This effect may be a result of the cationic character of this peptide, with interactions favored by the negatively charged sialic acid molecules present on the erythrocyte membranes [42,43] and by a net negative charge due to higher contents of anionic molecules (such as phosphatidylserine, O-glycosylated mucins, sialylated gangliosides and heparin sulfate) on cancer cells [44,45], as well as its highly hydrophobic feature. However, several strategies have been proposed to increase selectivity, decreasing the hemolytic activity. Such strategies include residue substitution/incorporation with natural and/or unnatural amino acids, C- and N-terminal modification, cyclization, dimerization, l- to d- amino acid isomerization, incorporation with nanoformulations/nanoparticles, and the computer-aided design of AMPs using artificial intelligence technology to overcome the deleterious effects of some AMPs on host tissues [46,47,48,49,50]. An interesting example of the use of these technologies was the production of analogs of the hybrid antimicrobial peptide CA-MA, derived from two natural AMPs: residues 1 to 8 from Cecropin A (isolated from the hemolymph of *Hyalophora cecropia*, the giant silk moth) and residues 1 to 12 of Magainin 2 (isolated from the skin secretion of *Xenopus laevis*, the African clawed frog). The CMA3 analog showed strong antimicrobial activity (including activity against drug-resistant strains of *Escherichia coli* and *Pseudomonas aeruginosa*) and little cytotoxicity toward human red blood cells (hRBCs) or HaCaT cells [51]. In another example, Cao et al. modified the peptide Hylin a1 with the conjugation of an RGD tripeptide (Arg–Gly–Asp) to improve its activity against tumor cells and loaded the peptide RGD-Hylin a1 onto mesoporous silica (HMS), as a pH-dependent delivery system, forming a nano-system: RGD-Hylin-a1-HMS. This approach significantly reduced hemolytic activity and showed excellent cytotoxic and anti-tumor activities in vitro and in vivo without side effects [52]. Such strategies can be used in order to increase the selectivity of Figainin 1 for cancer cells (and reduce its adverse effects on noncancerous cells), thus enabling its use as a possible therapeutic agent.

## 5. Conclusions

In summary, herein, we reported the purification and characterization of Figainin 1, a novel antimicrobial and antiproliferative peptide isolated from the cutaneous secretion of the frog *Boana raniceps* (Anura: Hylidae). This peptide is cationic, C-terminally amidated and rich in hydrophobic amino acids and adopts an α-helical conformation in the presence of TFE, a hydrophobic membrane-mimetic environment. Figainin 1 shows activity against Gram-negative and Gram-positive pathogenic bacteria in addition to epimastigote forms of *T. cruzi* but is inactive against *Candida* species. It also has potent cytolytic activity against human erythrocytes and antiproliferative effects on cancer cell lines. Despite toxicity in its native form, Figainin 1 represents a potential lead molecule for the development of new anticancer agents and anti-infective drugs against pathogenic Gram-positive bacteria.

## Figures and Tables

**Table 1:** Biophysical properties of Figainin 1.

Peptide	MM (calc) ^a^ (Da)	MM (obs) ^b^ (Da)	Net Charge	Hydrophobic Ratio (%)	GRAVY
Figainin 1	1915.19	1914.20	+3	61	1.46

^a^ MM (calc) is the calculated monoisotopic molecular mass based on the proposed structure of Figainin 1. ^b^ MM (obs) is the observed monoisotopic molecular mass of the deprotonated form of Figainin 1.

**Table 2:** Minimal inhibitory concentration (MIC, µM and g/L) for representative pathogenic microorganisms, and half maximal inhibitory concentration (IC_50_, µM and g/L) against epimastigote forms of *T. cruzi* displayed by Figainin 1.

	Figainin 1	
Microorganisms	µM	g/L
**Gram-positive bacteria (MIC)**		
*E. faecalis* (ATCC 29212)	8	0.015
*S. aureus* (ATCC 25923)	4	0.008
*S. epidermidis* (ATCC 12228)	2	0.004
*E. casseliflavus* (ATCC 700327)	16	0.030
**Gram-negative bacteria (MIC)**		
*E. coli* (ATCC 25922)	16	0.030
*P. aeruginosa* (ATCC 27853)	NA ^a^	NA ^a^
*K. pneumoniae* (ATCC 13883)	4	0.008
**Yeast (MIC)**		
*C. albicans* (ATCC 90028)	NA ^a^	NA ^a^
*C. parapsilosis* (ATCC 22019)	NA ^a^	NA ^a^
***Trypanosoma* epimastigotes (IC_50_)** *T. cruzi*	15.9	0.030

^a^ NA: no activity observed at a maximum concentration of 64 µM (0.123 g/L).
